# Synergistic Effects of *GhSOD1* and *GhCAT1* Overexpression in Cotton Chloroplasts on Enhancing Tolerance to Methyl Viologen and Salt Stresses

**DOI:** 10.1371/journal.pone.0054002

**Published:** 2013-01-15

**Authors:** Xiaoli Luo, Jiahe Wu, Yuanbao Li, Zhirun Nan, Xing Guo, Yixue Wang, Anhong Zhang, Zhian Wang, Guixian Xia, Yingchuan Tian

**Affiliations:** 1 State Key Laboratory of Plant Genomics, Institute of Microbiology, Chinese Academy of Sciences, Beijing, China; 2 Institute of Cotton Research, Shanxi Academy of Agricultural Sciences, Yuncheng, China; New Mexico State University, United States of America

## Abstract

In plants, CuZn superoxide dismutase (CuZnSOD, EC l.15.1.1), ascorbate peroxidase (APX, EC 1.11.1.11), and catalase (CAT, EC l.11.1.6) are important scavengers of reactive oxygen species (ROS) to protect the cell from damage. In the present study, we isolated three homologous genes (*GhSOD1*, *GhAPX1*, and *GhCAT1*) from *Gossypium hirsutum*. Overexpressing cassettes containing chimeric *GhSOD1*, *GhAPX1*, or *GhCAT1* were introduced into cotton plants by *Agrobacterium* transformation, and overexpressed products of these genes were transported into the chloroplasts by transit peptide, as expected. The five types of transgenic cotton plants that overexpressed *GhSOD1*, *GhAPX1*, *GhCAT1*, *GhSOD1* and *GhAPX1* stack (SAT), and *GhSOD1* and *GhCAT1* stack (SCT) were developed. Analyses in the greenhouse showed that the transgenic plants had higher tolerance to methyl viologen (MV) and salinity than WT plants. Interestingly, SCT plants suffered no damage under stress conditions. Based on analyses of enzyme activities, electrolyte leakage, chlorophyll content, photochemical yield (*Fv/Fm*), and biomass accumulation under stresses, the SCT plants that simultaneously overexpressed *GhSOD1* and *GhCAT1* appeared to benefit from synergistic effects of two genes and exhibited the highest tolerance to MV and salt stress among the transgenic lines, while the SAT plants simultaneously overexpressing *GhSOD1* and *GhAPX1* did not. In addition, transgenic plants overexpressing antioxidant enzymes in their chloroplasts had higher tolerance to salt stress than those expressing the genes in their cytoplasms, although overall enzyme activities were almost the same. Therefore, the synergistic effects of *GhSOD1* and *GhCAT1* in chloroplasts provide a new strategy for enhancing stress tolerance to avoid yield loss.

## Introduction

Throughout their life cycles, higher plants are exposed to conditions unfavorable to their growth and development, particularly in continuously-changing environments. These abiotic stresses can lead to plant injury and death. Reactive oxygen species (ROS) are generated in excess during abiotic stress; they are a major source of damage in plants, because they cause oxidative damage at the cellular level [1]–[3]. The ROS, including superoxide anion radical (O_2_^–^), hydrogen peroxide (H_2_O_2_), and hydroxyl radicals (OH^–^), are usually formed at low level within cells as byproducts of normal metabolic reactions [4]. However, increased production of ROS occurs when cell physiological homeostasis is disrupted by certain stresses, such as excess light, extreme temperatures, water deficit, metallic toxicity, salinity, and wounding [1], [2], [5], [6], [7], [8]. The ROS are extremely reactive in nature because they can interact with a number of other molecules and metabolites, such as DNA, pigments, proteins, lipids, and other essential cellular molecules, and subsequently lead to a series of destructive processes [9], [10].

The damaging effects of ROS have caused plant cells to cope with oxidative stress by triggering complex redox homeostatic antioxidative mechanisms. These ROS-scavenging antioxidative mechanisms include specific antioxidant enzymes, such as superoxide dismutase (SOD), ascorbate peroxidase (APX), peroxidase, catalase (CAT), and some other low-molecular-weight antioxidants [3], [5], [11]. Some studies have demonstrated that the antioxidant enzymes comprise several isoenzymes located in different cellular compartments of higher plants, such as the cytosol, chloroplasts, microbodies, and mitochondria, indicating their important roles in controlling cellular ROS levels in multiple stress responses [12]. The chloroplast, an important plant organelle with a high-energy photosynthetic electron transport system and a generous supply of oxygen, is a rich source of ROS [5]. Two key ROS antioxidant enzymes in the chloroplast are SOD and APX. SOD first catalyzes the dismutation of two O_2_^–^ into O_2_ and H_2_O_2_, and then APX uses ascorbate as an electron donor to reduce H_2_O_2_ to water [5], [13]. In the leaves of higher plants, H_2_O_2_ is also generated in bulk in peroxisomes during photorespiration [14]. CAT is also an essential enzymatic system used to degrade H_2_O_2_ into water, thereby lowering intracellular H_2_O_2_ levels [15]–[17].

Plants overexpressing these scavenging enzymes had been engineered with the goal of enhancing protection against stresses. In most cases, these transgenic plants exhibited increased tolerance to various stresses, such as ozone [18], extreme temperature [13], [19]–[21], salinity [9], [22], [23], methyl viologen (MV) [19], [24]–[28], and other stresses [29]–[31]. In a few cases, these plants did not show the increased stress tolerance [32], [33]. Ashraf and Harris [34] suggested that stress tolerance is a multigenic trait that cannot be substantially increased by introducing any single gene. Recently, some reports have shown that transgenic plants expressing multiple genes have greater tolerance to various environmental stresses than those expressing single genes [6], [13], [35]–[38]. However, Payton et al. [39] found no synergistic enhancement of stress tolerance in transgenic cotton hybrids overexpressing SOD and APX. Shikanai et al. [40] suggested that under stress conditions, APXs were completely inactivated in tobacco; however, the CAT still exhibited high activity in the chloroplasts and could reduce H_2_O_2_ instead of APX. Thus, previous attempts to produce stress-tolerant plants have mainly focused on the manipulating a single scavenging enzyme or APX in combination with other antioxidants; data on effects of simultaneous expression of CAT and other antioxidants, especially in chloroplasts, are lacking. Thus, an interesting question is whether the ROS level in whole plant could be controlled by reducing ROS production in chloroplasts via overexpression of SOD and CAT. Ideally, this strategy could provide good protection against ROS for transgenic plants exposed to stress conditions.

In this study, we investigate the synergistic effects on stress tolerance of expressing both CAT and SOD in chloroplasts. *GhSOD1* (GenBank accession number: DQ445093), *GhAPX1* (EF432582.1), and *GhCAT1* (X52135) were isolated from *Gossypium hirsutum*, and overexpressed in cotton plants under control of the CaMV35S promoter. Overexpressed products were transported by transit peptide into the chloroplasts, where they accumulated. Two new types of transgenic cotton plants co-expressing either *GhSOD1* and *GhCAT1* or *GhSOD1* and *GhAPX1* were produced by cross-pollination. The transgenic plants co-expressing *GhSOD1* and *GhCAT1* in chloroplasts exhibited higher tolerance to MV and salinity stresses than other transgenic plants with single genes or with *GhSOD1* and *GhAPX1*. These results indicated that GhCAT1 can scavenge H_2_O_2_ when GhAPX1 is inactivated under stress conditions and that the combination of *GhSOD1* and *GhCAT1* targeted in chloroplasts may help cotton withstanding stresses, information that could guide the development of transgenic cotton cultivars for increased stress tolerance.

## Results

### Isolation and Characterization of *GhSOD1, GhAPX1, and GhCAT1*

About 500 clones containing salt stress-regulated genes from cotton were obtained through Suppression Subtractive Hybridization (SSH). Sequencing some clones identified three expressed sequence tag (EST) sequences that were highly homologous with *Cu/Zn SOD1*, *APX1*, and *CAT1* in *Arabidopsis*. To clone the full-length cDNA of the three genes, RT-PCR was performed using primers specific to previously-identified ESTs. The cloned genes were termed *GhSOD1*, *GhAPX1*, and *GhCAT1* and submitted to GenBank under accession numbers DQ445093, EF432582.1, and X52135, respectively. *Gossypium hirsutum* is an allotetroploid, with At and Dt subgenome. To further characterize the three genes, we searched for homologs in the *Gossypium raimondii* (a diploid species, D5 subgenome) genome database using BLAST. The highest nucleotide identities of *GhSOD1*, *GhAPX1*, and *GhCAT1* in *G. raimondii* were to GR__Ea06I18 (GenBank no. CO087877; 97.62% identity), GR__Ea21L06 (CO097712; 98.54%), and GR__Ea48E14 (CO083282; 99.04%) genes, respectively. These results indicated that the three genes could have two copies, one residing in each subgenome of *G. hirsutum*.

Tissue-specific expression levels of *GhSOD1*, *GhAPX1*, and *GhCAT1* in cotton plants were determined by semi-quantitative RT-PCR. Transcriptional expression of all three genes was detected in roots, stems, leaves, cotyledons, petals, anthers, and ovules (Fig. S1). However, their transcription levels were higher in some tissues (*GhSOD1* in leaf, stems, and ovules; *GhAPX1* in leaves and roots; and *GhCAT1* in leaves and ovules), suggesting their specific roles in these tissues.

### PCR and Southern Blot Analyses of the Transgenic Cotton Plants

**Figure 1:**
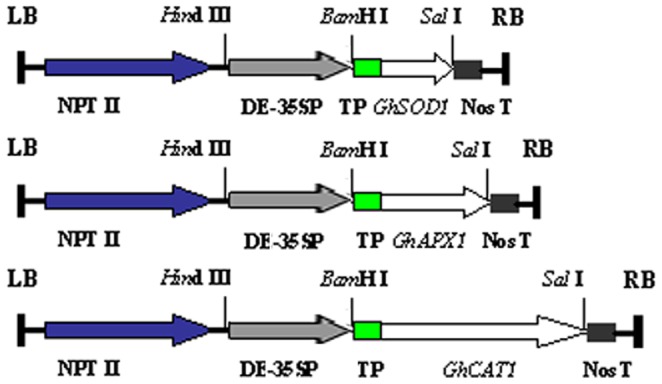
Schematic diagrams of the T-DNA structure of the plant expression vectors. *CaMV35S::GhSOD1* (top), *CaMV35S::GhAPX1* (middle), and *CaMV35S::GhCAT1* (bottom) constructs. NPT II, neomycin phosphotransferase II; DE-35SP, CaMV35S promoter with double-enhancer sequence; TP, transit signal peptide; Nos T, transcriptional termination sequence of nopaline synthase gene; LB, left border of T-DNA; RB, right border of T-DNA.

*CaMV35S::GhSOD1*, *CaMV35S::GhAPX1*, and *CaMV35S::GhCAT1* constructs were produced (Fig. 1). Transgenic cotton plants overexpressing *GhSOD1*, *GhAPX1*, or *GhCAT1* in chloroplasts were developed by the *Agrobacterium*-mediated transformation. At least 50 individual kanamycin-resistant transgenic plants were obtained for each vector and subjected to PCR with specific primers (Table S1). To avoid interference from endogenous genes in cotton, the forward primer was located in the *thi1*chloroplast transit signal sequence from *Arabidopsis*, and the reverse primers were positioned in the *GhSOD1*, *GhAPX1*, or *GhCAT1* genes (Table S1). Specific bands were amplified from transgenic cotton plants that corresponded in size to the constructs *CaMV35S::GhSOD1*, *CaMV35S::GhAPX1*, and *CaMV35S::GhCAT1,* whereas no bands were obtained from control plants (Fig. S2A). A total of 41 plants had *CaMV35S::GhSOD1*, 37 had *CaMV35S::GhAPX1*, and 43 had *CaMV35S::GhCAT1.*

**Figure 2:**
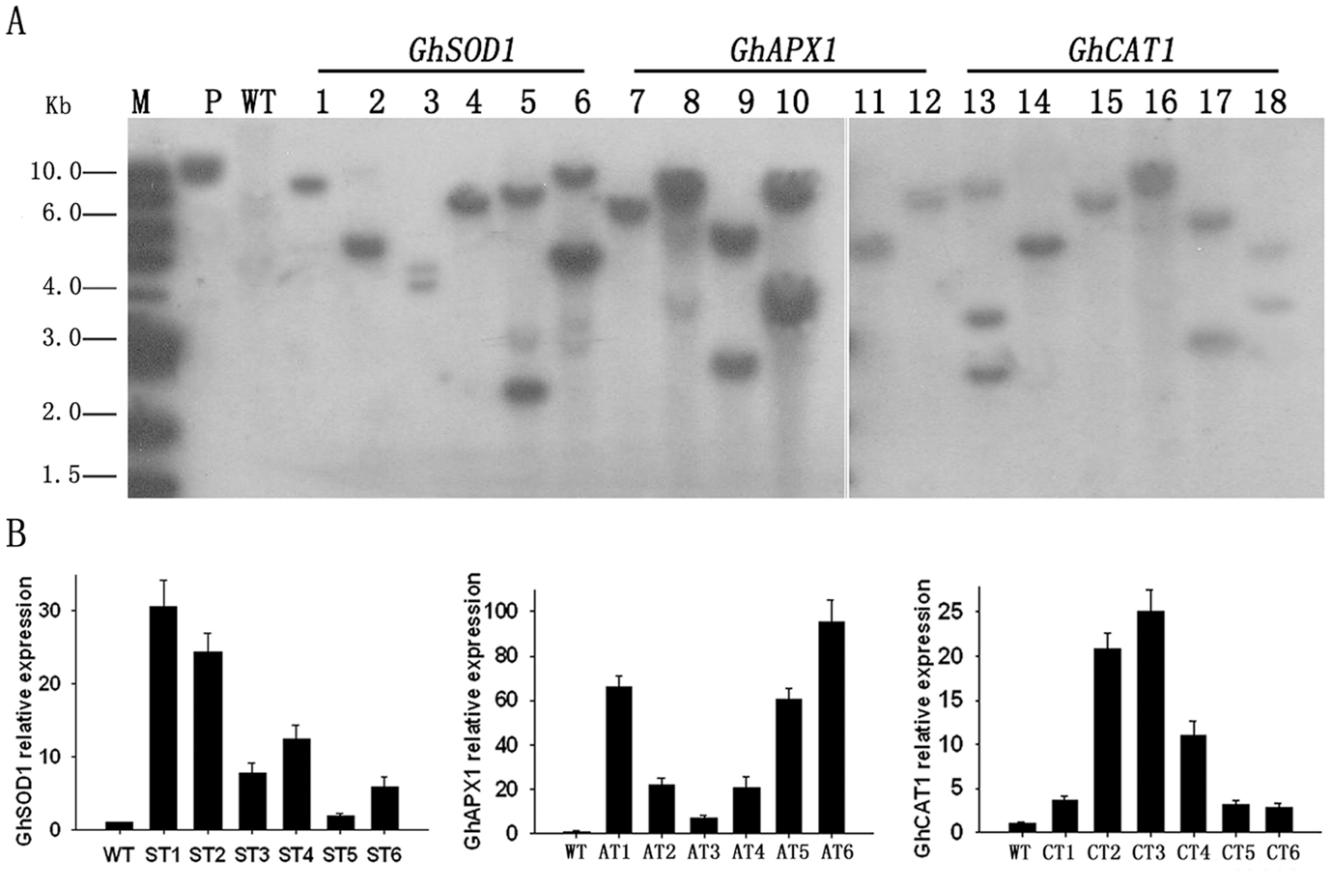
Southern blot and qRT-PCR analysis of transgenic cotton overexpressing *GhSOD1*, *GhAPX1*, or *GhCAT1*. (A) Southern blot. M, DNA marker; P, pBin438 plasmid; 1–6, representative transgenic plants with *CaMV35S::GhSOD1* (1, 2, and 4 are listed as ST1, ST2, and ST4 below); 7–12, representative transgenic plants with *CaMV35S::GhAPX1* (1, 5, and 6 are listed as AT1, AT5, and AT6 below); 13–18, representative transgenic plants with *CaMV35S::GhCAT1* (2, 3, and 4 are listed as CT2, CT3, and CT4 below). DNA of the pBin438 plasmid and transgenic and WT plants was digested with *Bam*HI (only one *Bam*HI restriction site is present within the T-DNA region). The *npt II* gene-specific probe was PCR-amplified from pBin438 vector. (B) qRT-PCR analysis. Values are given as means ± standard deviation.

To ascertain how many copies of the constructs were inserted, the PCR-positive plants were further analyzed by Southern blot using *NPT II* as a probe. The results showed that the transgenic plants contained 1–3 copies of the *CaMV35S::GhSOD1*, *CaMV35S::GhAPX1*, or *CaMV35S::GhCAT1* constructs (Fig. 2A). Plants with single insertion copies and no obvious phenotypic changes were chosen for further functional analyses.

### Semi-quantitative RT-PCR and Quantitative Real Time RT-PCR Analyses of Transgenic Cotton Plants

To examine the transcription levels of *GhSOD1*, *GhAPX1*, and *GhCAT1* in transgenic cotton plants, semi-quantitative RT-PCR was performed using the PCR primers described above. Some PCR-positive plants exhibited specific RT-PCR bands of the same size as the PCR products of the plasmids containing the corresponding target genes, whereas WT plants yielded no bands (Fig. S2B). Overall, transcriptional expression was detected in 33 plants with *CaMV35S::GhSOD1*, 28 plants with *CaMV35S::GhAPX1*, and 31 plants with *CaMV35S::GhCAT1*.

Based on the combined results of Southern blot and semi-quantitative RT-PCR analyses, we selected six independent transgenic plants with single-copy insertions and high transcriptional levels for each construct. We evaluated their transcriptional levels by quantitative real time RT-PCR (qRT-PCR). In transgenic plants, *GhSOD1*, *GhAPX1*, and *GhCAT1* were transcribed at levels that were 1.9–30.6, 7.3–95.2, and 3.5–24.5 fold those in WT plants, respectively (Fig. 2B). Three plants per construct (ST1, ST2, and ST4 for *GhSOD1*; AT1, AT5, and AT6 for *GhAPX1*; and CT2, CT3, and CT4 for *GhCAT1*) were selected for further investigations.

To evaluate the tolerances of transgenic cotton plants, especially those with two-gene stacks, to various stresses, homozygous lines with single-copy insertions, high transcription levels, and no obvious phenotypic changes were used to produce two-gene stack (*GhSOD1*/*GhAPX1* or *GhSOD1/GhCAT1*) plants by cross pollination. qRT-PCR analyses showed that plants with two-gene stacks exhibited the same high transcriptional levels of target genes as their parents (data not shown). Thereafter, five homozygous types of transgenic cotton, namely ST, AT, CT, SAT, and SCT were developed. These five types respectively overexpressed *GhSOD1*, *GhAPX1*, *GhCAT1*, *GhSOD1*+ *GhAPX1* stack, and *GhSOD1*+ *GhCAT1* stack. A total of 15 homozygous lines (three per type) of transgenic cotton were further characterized. These 15 lines were ST1, ST2, ST4, AT1, AT5, AT6, CT2, CT3, CT4, SAT1, SAT2, SAT4, SCT1, SCT2, and SCT4.

### Enzyme activity of the Transgenic Cotton Plants

**Figure 3:**
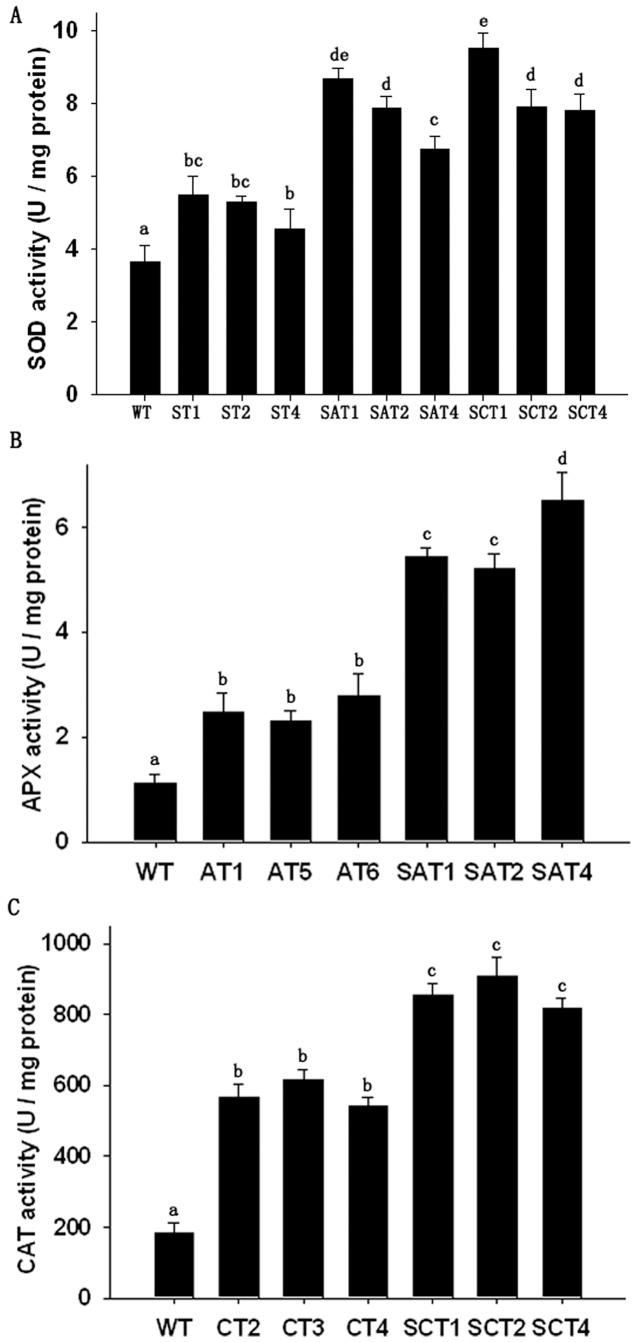
Activities of antioxidant enzymes in leaves of transgenic and WT cotton plants. (A) SOD activity. (B) APX activity. (C) CAT activity. Means ± standard deviation labeled with different letters are significantly different at the 0.05 level.

To reveal whether the enzyme activities of SOD, APX, and CAT in transgenic plants had increased, we analyzed enzyme activities in leaves of transgenic plants overexpresing *GhSOD1*, *GhAPX1*, and *GhCAT1* alone and in two-gene combinations. In comparison with WT plants, the average SOD activity was 1.6-fold in ST, 2.4-fold in SAT, 2.6-fold in SCT (Fig. 3). The APX activity of SAT plants was 2.1-fold that of AT plants, and 5.4-fold that of WT plants. The CAT activities of CT and SCT plants were 3.2- and 5.3-fold that of WT plants. These results suggested that transgenic plants that overexpressed two antioxidant genes (SAT and SCT) had higher activities of corresponding enzymes than did transgenic plants overexpressed only one antioxidant gene (ST, AT, and CT lines). The increase in total SOD, APX, and CAT activities further confirmed that they were overexpressed in transgenic plants.

**Figure 4:**
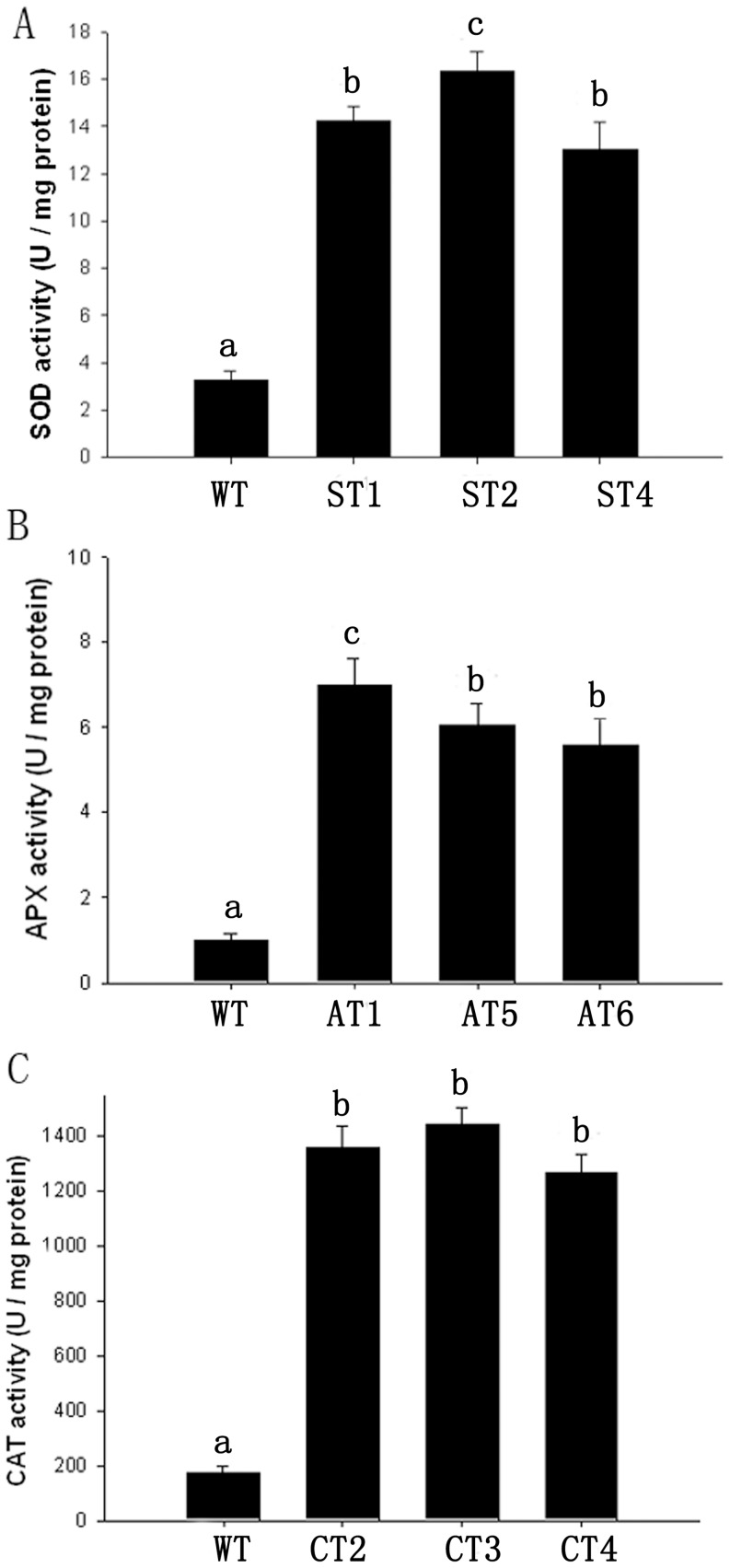
Activities of antioxidant enzymes in chloroplasts of the transgenic and WT cotton plants. (A) SOD activity. (B) APX activity. (C) CAT activity. Means ± standard deviation labeled with different letters are significantly different at the 0.05 level.

The activities of three enzymes in chloroplasts were further assayed. The average activities of SOD, APX, and CAT in chloroplasts of ST, AT, and CT plants were significantly higher than those in WT plants, by 4.4-, 6.3-, and 7.7- fold respectively (Fig. 4), which indicated that overexpression of *GhSOD1*, *GhAPX1*, and *GhCAT1* had been targeted in the chloroplasts.

### Increased Tolerance of Transgenic Cotton Plants to MV-induced Oxidative Stress

**Figure 5:**
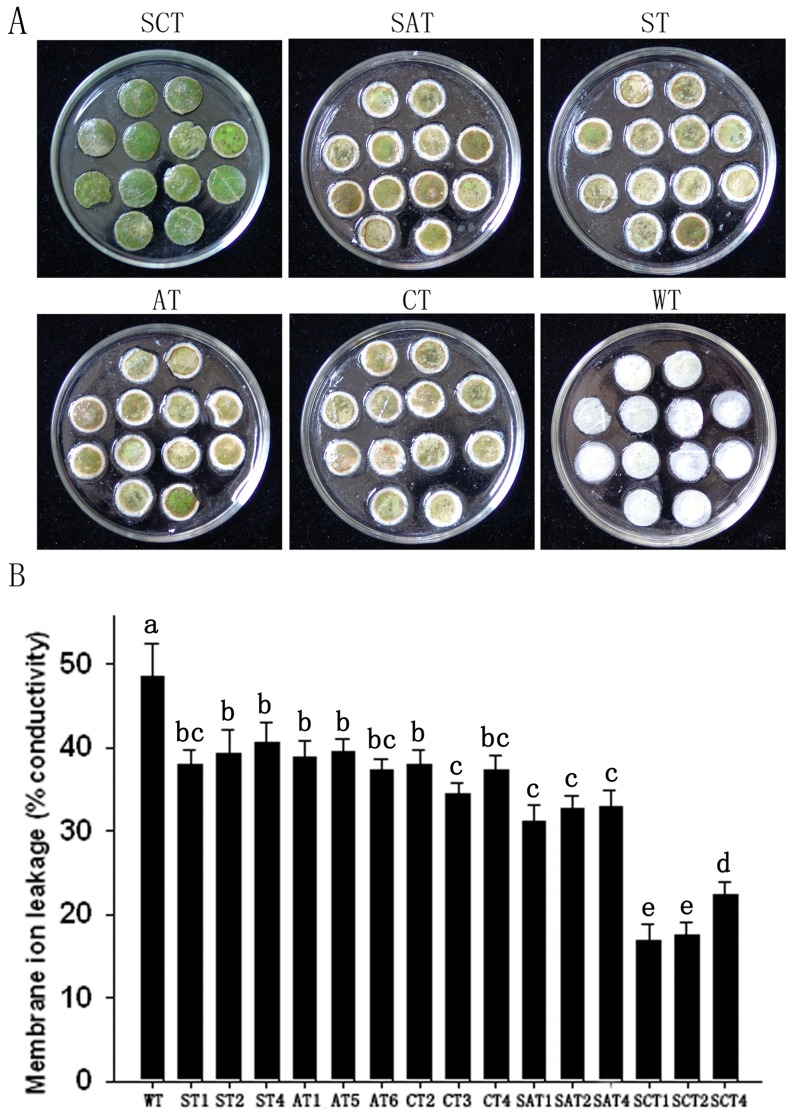
Enhanced MV-mediated oxidative stress tolerance in leaf discs of transgenic cotton lines. (A) Visible damage in leaf discs of WT and transgenic plants 36 h after 5 µM MV treatment. (B) Relative electrolyte leakage assay. Means ± standard deviation labeled with different letters are significantly different at the 0.05 level.

To assess the tolerance to oxidative stress, leaf discs of five types of transgenic and WT plants were incubated with 5 µM MV under illumination. MV is a typical ROS-generating redox-active compound and, as a non-selective herbicide, destroys chlorophyll synthesis and chloroplast formation [41]. The extent of leaf-disc damage in the transgenic lines 36 h after MV treatment was substantially reduced compared with WT. Severe damage occurred in WT leaf discs, whereas only partial necrosis around the disc edges was observed in ST, AT, CT, and SAT plants. Interestingly, the leaf discs from SCT plants remained green, with no observed damage (Fig. 5A). The loss of cytoplasmic solution in leaf discs 48 h after treatment with 5 µM MV was determined by monitoring the electrical conductance of the solution. The transgenic lines had significantly lower levels of ion leakage than the WT control (Fig. 5B). The SCT1 (the best SCT line) showed the least ion leakage (16.8%), while the leakages of ST1, AT6, CT3, SAT1 (each the best line of its type), and WT were, respectively, 38.0, 37.4, 34.5, 31.1, and 48.4%. Thereby, the SCT1 showed the highest tolerance to MV, although other transgenic lines exhibited moderate tolerance.

**Figure 6:**
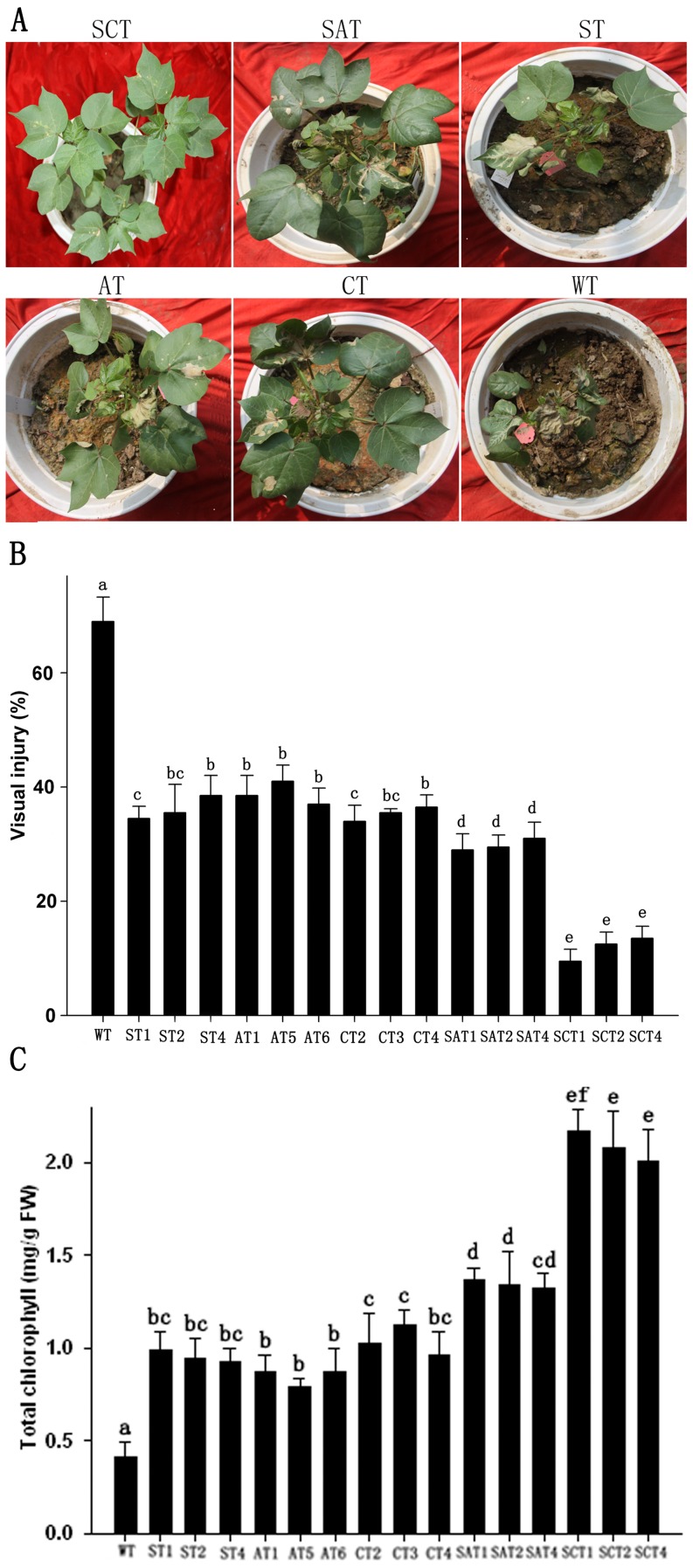
Enhanced MV-mediated oxidative stress tolerance in whole plants of transgenic and WT cotton. (A) Visible damage in leaves with 300 µM MV for 5 d. (B) Quantitative estimate of visible damage on leaves. (C) Total chlorophyll contents in MV-treated transgenic plants. Means ± standard deviation labeled with different letters are significantly different at the 0.05 level.

The tolerance of the transgenic plants to oxidative stress was also investigated at the whole-plant level. The WT plants sprayed with 300 µM MV exhibited obvious leaf damage, while the transgenic lines showed significantly less leaf damage, especially SCT, which were almost unaffected (Fig. 6A). The WT plants showed 68% visible leaf damage, whereas the SCT1 plants only had an average of 9% visible leaf damage; values for ST1, AT6, CT3, and SAT1 were 35, 37, 36, and 30%, respectively (Fig. 6B). To further evaluate the tolerance of transgenic lines to MV, the total chlorophyll content was tested. The result confirmed that the SCT plants were significantly more tolerant to MV than WT and other transgenic plants (Fig. 6C). These results suggested that the SCT plants were more tolerant to oxidative stress than the WT and other transgenic plants.

### Increased Tolerance of Transgenic Cotton Plants to Salt Stress

**Figure 7:**
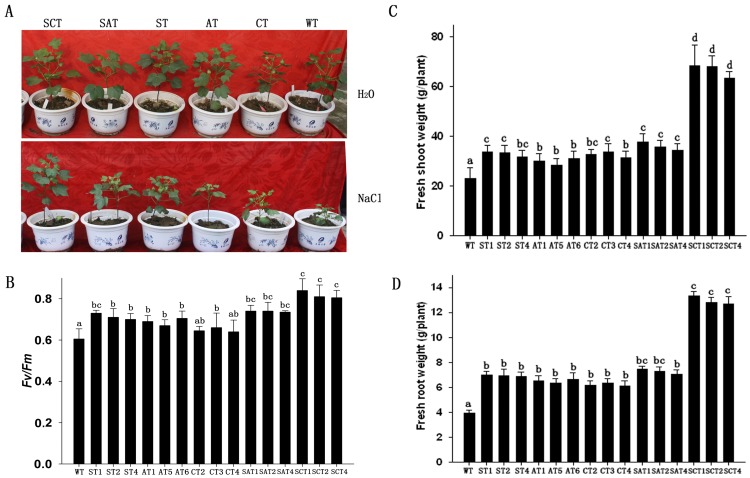
Increased tolerance of salt stress in transgenic cotton plants. (A) Phenotypes of WT and transgenic plants 21 d after treatment with 200 mM NaCl. Plants shown were 9 wks old. (B) Photosynthetic *Fv/Fm* of leaves of transgenic and WT plants 2 d after treatment with 200 mM NaCl. (C and D) Fresh shoot (C) and root (D) biomasses of transgenic and WT plants after treatment with 200 mM NaCl for 4 wks. Means ± standard deviation labeled with different letters are significantly different at the 0.05 level.

In plants, ROS can be usually induced under abiotic stresses, e.g., salinity, high light, extreme temperature, water deficit. Healthy, 3-wk-old plants of WT and five types of transgenic cotton were treated with salt in an incremental manner. Another group of plants was treated with tap-water as a control, and all of these plants grew similarly. We gradually increased the salt concentration to acclimate the plants to salt stress, and our transgenic and control plants could both flower and set seed. However, dramatic phenotypic differences were observed among WT ans five types of transgenic plants when the salt concentration increased to 200 mM NaCl for 3 wks. The growth of WT was severely inhibited (plants were dwarfed, with small leaves), whereas SCT grew similarly to the control plants and the other four types of transgenic plants exhibited partially-inhibited growth (Fig. 7A). In addition, to evaluate function of antioxidant enzymes accumulated in chloroplasts, the transgenic plants overexpressing both *GhSOD1* and *GhCAT1* in the cytoplasm (SCT-p, without chloroplast targeting) were produced. The SCT-p plants exhibited lower tolerance to salt stress than the SCT (SCT-c in Fig. S3) plants, although their antioxidant enzyme activities were almost identical.

To evaluate changes in photosynthetic activity in salt-stressed plants, the fluorescence parameters (*Fv/Fm*) of leaves from the WT and transgenic lines were measured. Salt stress affected the photosynthetic machinery of plants treated by 200 mM NaCl for 2 d; however, the transgenic SCT plants maintained significantly higher levels of photosynthetic activity than the other transgenic or WT plants, and the other transgenic lines also exhibited significantly higher activity than WT (Fig. 7B).

To evaluate the biomass differences among transgenic and WT plants, fresh shoot and root masses were analyzed. The values for SCT plants were significantly higher (3.0- and 3.4-fold, respectively) than in WT plants. Fresh shoot and root masses of the other transgenic plants were also significantly higher than those of WT plants (Fig. 7C,D). Plant height, bolls per plant, and yield per plant were highest in the SCT lines, although those for the other transgenic lines were also significantly higher than WT (Table 1). In the control (tap-water) group, the transgenic plants had similar traits to the WT plants, with no significant differences in agronomic characters between transgenic lines and WT (Table S2).

**Table 1:** Agronomic traits of transgenic and WT cotton plants treated with 200 mM NaCl in the greenhouse.

Lines	Plant height (cm)	Bolls per plant	Boll weight (g)	Seed fiber yields per plant (g)
WT	39.2±3.8 a	3.8±0.4 a	4.8±0.4 a	18.3±2.5 a
ST	61.8±6.1 b	6.7±0.5 b	4.9±0.3 a	32.9±3.0 b
AT	60.5±4.2 b	6.4±0.5 b	4.7±0.5 a	30.1±2.6 b
CT	59.8±6.2 b	6.2±0.4 b	5.0±0.5 a	30.9±2.8 b
SAT	64±6.4 b	6.9±0.6 bc	4.9±0.2 a	33.9±3.6 b
SCT	78.5±8.2 c	8.6±0.8 c	5.1±0.4 a	44.0±4.1 c

Three lines each of five types of transgenic cotton (six plants per line, 18 WT plants) were evaluated; the experiment was repeated three times. Values are given as means ± standard deviation (n = 54). Means within a column followed by different letters are significantly different at *P*<0.05.

## Discussion

How to feed and clothe the growing population is a major challenge of our century because world crop production has not been increasing at a rate comparable with the population increase [42]. Therefore, new strategies and technologies to make crops more productive in stressful environments are imperative. Genes have been identified that could be used to enhance crop yield and quality [43]. Among them, *SOD*, *APX*, and *CAT* have been introduced into many crops to improve stress tolerance [6], [13], [15], [18], [20], [22]–[25], [27]–[30], [35]–[38]. In this study, we isolated *GhSOD1*, *GhAPX1*, and *GhCAT1*from cotton and produce five types of transgenic cotton plants overexpressing theses genes individually or in combination in chloroplasts. All of the transgenic plants had enhanced tolerance to MV and salinity stresses, but simultaneous overexpression of *GhSOD1* and *GhCAT1* in chloroplasts conferred the highest tolerance.

In recent years, vast amounts of the available land have been affected by salinity, which further decreases the acreage available for crop production [44]. Today, about 20% of the world’s cultivated land and nearly half of all irrigated fields are affected by salinity [45], [46]. In China, about 37 million ha of land has high or moderate salinity and cannot be used for crop production [47]. Salt stress triggers oxidative stress in plant tissues; salt stress could reduce gas exchange and limit CO_2_ supply to the leaf [5], which causes an over-reduction of the photosynthetic electron transport chain [48] and concomitant production of ROS such as singlet oxygen (^1^O_2_), O_2_^−^, H_2_O_2_, and OH^-^ [5], [49]. Thus, excessive ROS induced by salt stress must be removed in time to avoid plants suffered serious oxidative damage although they may also signal the induction of protection mechanisms [6], [50], [51]. In this study, we gradually increase the salt concentration to acclimate the plants to salt stress for cotton moderate tolerance [52], and our transgenic and control plants could flower and set seed. We found that transgenic plants were significantly more tolerant to oxidative stress caused by MV and had higher growth and fiber yield under salt stress than WT plants. Transgenic SCT plants overexpressing *GhSOD1* and *GhCAT1* showed no damage under MV treatment and had the highest growth and fiber yields under salt stress (Fig. 6 and 7). Consistent with our results, many reports on transgenic plants that overexpressed *SOD*, *CAT*, and *APX* found increased tolerance to MV [9], [19], [24]–[28] and salinity stress [9], [22], [23].

Simultaneous overexpression of *GhSOD1* and *GhAPX1* in this study caused no synergistic effects on MV or salt tolerance. Payton et al. [39] also reported that transgenic cotton hybrids with both *SOD* and *APX* exhibited no synergistic effects on enhanced tolerance to environmental stresses. Shikanai et al. [40] suggested that under the stress conditions, *APX* were completely inactivated in the plants and that high CAT high activity reduced H_2_O_2_ instead of APX in the chloroplasts. Under the oxidative stress, excess H_2_O_2_ generation may deplete reduced ascorbate, which inactivates APX [21], [53], [54]. Thus, the ascorbate-glutathione cycle may function only under mild environmental conditions. We also observed that the total APX activity of transgenic plants 2 d after treatment with 200 mM NaCl was only 31.5% of that in control plants, whereas CAT activity of transgenic plants treated with whether salt or H_2_O was almost same (data not shown). These results implied that salt stress inactivated the ascorbate-glutathione cycle in chloroplasts, injuring the plants. Our results confirmed that overexpressing both *GhSOD1* and *GhCAT1* in chloroplasts could synergistically affect ROS scavenging in plant cells and thus confer enhanced tolerance to environmental stress.

Transgenic plants overexpressing *SOD*, *APX*, or glutathione reductase (*GR*) targeted into chloroplasts had increased tolerance to oxidative stress [13], [18], [39], [55], chilling stress [21], salinity stress [22], [23], and MV [24], [25], [27], [28] in cotton and other plants. However, others studies found no increased tolerance to stress in transgenic cotton plants [32], [33], [56]. These different results might attribute to the complexity of the ROS detoxification system, because regulating a single enzyme activity may not change the capacity of the pathway as a whole [34], [46]. This hypothesis suggests that expressing combinations of antioxidant enzymes in transgenic plants might have synergistic effects on stress tolerance. Thus far, the few reports that have examined the expression of two or more enzymes in the chloroplasts of higher plants described increased tolerance to various stresses [13], [24], [35]–[38], [51]. However, transgenic cotton plants overexpressing *SOD* and *APX* exhibit no synergistic increase in tolerance to environmental stresses [39]. Therefore, transgenic plants with a single scavenging enzyme or with APX in combination with other antioxidants were not protected from stress. We produced transgenic cotton plants that simultaneously overexpressed *GhSOD1* and *GhCAT1* and had exceptional tolerance to MV and salt stress, although transgenic plants with a single antioxidant enzyme or with *GhSOD1* and *GhAPX1* could tolerate only mild stress.

The chloroplast can produce substantial amounts of ROS as a result of their high-energy photosynthetic electron transport system and rich supply of oxygen [5].To avoid damage under stress condition, ROS in the chloroplast must be scavenged. Kwon et al. [36] considered that plants overexpressing antioxidant enzymes could more rapidly scavenge superoxide anion radicals and H_2_O_2_ at the sites of generation, prior to their interaction with target molecules. Therefore, many studies have employed different methods to transport the antioxidant enzymes to the chloroplast to lower intraorganellar ROS [13], [22], [23], [39], [55]. In this study, a chloroplast transit signal coding sequence of the *thi1* gene [57] was employed to target *GhSOD1*, *GhAPX1*, and *GhCAT1* in chloroplasts. The activities of SOD, APX, and CAT in chloroplasts of ST, AT, and CT plants, respectively, were significantly higher than in WT plants. The results suggested that the three antioxidant enzymes had been transported into the chloroplasts by signal peptide, as expected. In addition, the transgenic plants overexpressing *GhSOD1* and *GhCAT1* in cytoplasm (SCT-p) had significantly lower tolerance to salt stresses than those expressing the two genes in chloroplasts, although the activities of both antioxidant enzyme in leaves were almost the same. The function of THI1 signal peptide in cotton is consistent with that reported in *Arabidopsis* [57], who demonstrated that the transit signal peptide of THI1 protein could transfer the GFP protein into chloroplasts. In addition, we previously reported that Vip3A, a *Bacillus thuringiensis* toxin protein, was transferred into the chloroplasts of cotton by the transit signal peptide of *Arabidopsis* THI1 protein [58].

In addition, the transgenic plants that overexpressed two antioxidant enzymes had higher activities of SOD, APX, and CAT than the transgenic plants with single enzymes. Previous studies reported that transgenic plants that overexpressed *Cu/ZnSOD* had increased APX and CAT activities [59], [36]. Overexpressing *GhSOD1* may increase the native antioxidant enzyme activities (APX and CAT) in transgenic plants, further increasing the total activities of APX and CAT (native plus overexpression activities) in transgenic plants with multiple antioxidant enzymes. However, the mechanism should be further investigated.

In conclusion, the results presented in this study suggested that the transgenic cotton plants overexpressing antioxidant enzyme genes in chloroplasts showed enhanced tolerance to MV and salt stresses. These conclusions were supported by the better growth of and reduced damage to transgenic plants compared with the WT plants. In particular, plants simultaneously overexpresing *GhSOD1* and *GhCAT1* had the highest tolerances of the transgenic plants tested. Further experiments are required to evaluate performance of the transgenic cotton plants under other environmental stresses to further elucidate the individual and synergistic effects of antioxidant enzymes in cotton.

## Materials and Methods

### Plant Materials and Treatments

The cotton variety, *Gossypium hirsutum* L. cv. Zhongmiansuo 35, was used for genetic transformation. Zhongmiansuo35, which possesses good agronomic traits and is widely planted in China was kindly provided by Mr. Liu Zhengde from Henan Kerun Biotech. Co. (Henan province, Zhenzhou, China). Seeds were planted in 30 cm-diameter plastic pots filled with sterilized soil, and plants were grown in a greenhouse with natural lighting at 28/25°C (day/night) until maturation. The roots (25 d post-germination, DPG), stems (25 DPG), leaves (25 DPG), anthers (-1day after anthesis, DPA), petals (0 DPA), and ovules without fiber (7 DPA) were sampled, immediately frozen in liquid nitrogen, and stored at −80°C until use for RNA isolation.

### RNA Preparation, Gene Isolation, and Transcriptional Analysis

Total RNA was extracted from different organs/tissues using Trizol reagent according to the manufacture’s instructions (Invitrogen, Carlsbad, CA, USA) and treated extensively with RNase-free DNase I (Promega, Madison, WI, USA). First-strand cDNA was synthesized from 2 µg total RNA with the SuperScript™ First-Strand Synthesis system (Invitrogen). Of the cDNA obtained from reverse transcription (RT), 1 µl was polymerase chain reaction (PCR)-amplified to isolate the three target genes and investigate the transcriptional RNA level of the antioxidant enzyme genes, as described below.

The target genes were isolated on the basis of ESTs using SSH as proposed by Mathews et al [60]. The full-length cDNAs of *GhSOD1*, *GhAPX1*, and *GhCAT1* were produced by 5′ or 3′ rapid amplification of cDNA ends (RACE) using 5′- or 3′- RACE kits (Takara, Dalian, China). Finally, coding sequences (CDS) of the three genes were obtained by PCR amplification using various gene-specific primer pairs (Table S1) designed according to the sequences of RACE products. The amplified PCR products were ligated into the pGEM-T Easy Vector (Promaga) and positive clones were sequenced. All the primers were synthesized by Beijing Sunbiotech (Beijing, China). The PCR program was: 94°C for 3 min; followed by 30 cycles of 94°C for 30 s, 56°C for 50 s, and 72°C for 60 s; with a final extension step at 72°C for 10 min. Nucleotide and deduced amino acid sequences were analyzed using DNAMAN v. 5.2 (Lynnon Biosoft, Vandreuil, Quebec, Canada), and the clones were sequenced by Beijing Biosune (Beijing, China). The three genes were screened against the *Gossypium raimondii* (a diploid species, D5 subgenome) using BLAST to determine whether they resided in the At or Dt subgenome.

### Plasmid Construction and *Agribacterium*-mediated Cotton Transformation

A chloroplast transit-signal coding sequence from the thiamine biosynthetic gene (*thi1*) in *Arabidopsis* [57] was ligated to the 5′ ends of the CDSs of *GhSOD1*, *GhAPX1*, and *GhCAT1*. These chimeric genes were each inserted into the restriction enzyme sites of a binary vector pBin438 [61] to produce *CaMV35S::GhSOD1*, *CaMV35S::GhAPX1*, and *CaMV35S::GhCAT1* constructs (Fig. 1).

*Agrobacterium tumefaciens* LBA4404 harboring one of the three constructs was used to transform explants of *G. hirsutum* cv. Zhongmiansuo35. Transformation of cotton hypocotyl explants, initiation of callus, and regeneration of transformed plants were performed as described by Wu et al. [62]. Putative transgenic plants were transferred to the greenhouse within about 10 months. The T_0_ (original transgenic plant) seedlings were grown in soil for 3–4 months to set seed.

In addition, to evaluate the function of antioxidant enzymes accumulated in chloroplasts, transgenic plants overexpressing both *GhSOD1* and *GhCAT1* in the cytoplasm (SCT-p, without chloroplast targeting) were produced using methods described above for plant expression vector constructs and genetic transformation.

### PCR Detection and Southern Blot Analysis of Transgenic Plants

Genomic DNA was isolated from young leaves of transformed T_0_ and their offspring [63]. PCR was carried out using 20 ng of genomic DNA as template with gene-specific primers (Table S1) to amplify the transgene fragments. The amplicons were amplified by electrophoresis in agarose gels containing ethidium bromide. The PCR program was: 94°C for 3 min, followed by 30 cycles of 94°C for 30 s, 56°C for 40 s, and 72°C for 40 s, with a final extension step at 72°C for 10 min.

Southern blot analysis [64] was carried out using pBin438 vector DNA as a positive control. Approximately 20 µg of total genomic T_0_ DNA was digested with *Bam*HI, separated by electrophoresis on a 0.8% agarose gel, and transferred to a nylon membrane (Hybond-N+; Amersham Biosciences, Little Chalfont, UK). The sequence of *NPT II* was used as a probe labeled with α-[^32^P] using a random primer labeling kit (Promega).

### Transcription Level Analysis of Transgenic Plants

RNA isolation from leaves of transgenic and WT plants and cDNA syntheses were performed as described above. Then, 1 µl cDNA was amplified and expression of each gene was monitored by semi RT-PCR (see Table S1 for primers). To normalize the amount of cDNA, the internal control cotton *GhUBI* (accession number: EU604080) was amplified using the same cDNA as template. Amplicons were analyzed by electrophoresis in agarose gels containing ethidium bromide. Conditions for PCR were as described above.

To further quantify the target gene expression in the transgenic plants, qRT-PCR was employed. PCR assays were performed by using the SYBR Green Real-Time PCR Master Mix (Toyobo, Osaka, Japan) and the DNA Engine Opticon 2 Real-Time PCR Detection System (MJ Research, Hercules, CA, USA). The cotton *GhUBI* gene was used as an internal control to normalize expression. All reactions were performed in triplicate (samples from three different plants per transgenic line), and negative controls had no reverse transcriptase. Primer sequences are listed in Table S1. Data were processed to reveal relative transcript abundances using Opticon Monitor software (Bio-Rad, Hercules, CA, USA).

### Selection of Homozygous Transgenic Lines

In 2007, T_1_ progeny plants grown from seeds of T_0_ transformants with single-copy insertions, as determined by Southern blot analysis, were grown on 50% Murashige and Skoog (MS) medium [65] with kanamycin (200 mg L^−1^). Green plantlets with lateral root systems (10 plants per family) were transferred to soil and grown on the experimental farm of Institute of Cotton Research, Shanxi Academy of Agricultural Sciences, Yuncheng, China. Phenotypes were observed throughout the growth cycle, and mature seeds were harvested from T_1_ transgenic plants with normal phenotypes for generating T_2_ plants. Nine homozygous lines from three transgenic constructs (three lines per construct), namely ST1, ST2, ST4, AT1, AT5, AT6, CT2, CT3, and CT4, were selected based on the molecular and genetic analyses done in 2008. At the same time, transgenic cotton with different gene stacks (*GhSOD1*+ *GhAPX1* and *GhSOD1*+ *GhCAT1*) were produced by cross pollinating homozygous lines with high expressions of single target genes (ST1×AT6, ST2×AT6, and ST4×AT6, and ST1×CT3, ST2×CT3, and ST4×CT3, respectively). Three homozygous lines per combination, namely SAT1, SAT2, SAT4, SCT1, SCT2, and SCT4, were selected from self-pollinated populations based on molecular and genetic analyses of T_3_ plants in 2010. To speed the production of homozygous transgenic lines, the plants underwent two growth cycles per year, in summer in Shanxi Province and in winter in Hainan Province. Three homozygous lines from five transgenic germplasms (total 15 lines) with high target-gene transcription levels and no obvious phenotypic changes were selected and assayed for tolerance to MV and salt stress in the greenhouse.

### Analysis of Enzyme Activities in Leaves and Chloroplasts of Transgenic and WT Plants

Young, fully expanded leaves on the top of transgenic and WT plants in the greenhouse were collected to assay the total activities of SOD, APX, and CAT. Leaf tissue was ground in liquid nitrogen and suspended in homogenization buffer. The homogenization buffer for SOD and CAT was 50 mM potassium phosphate (pH 7.0) containing 0.1 mM EDTA, whereas for APX was 50 mM HEPES (pH 7.0) containing 1 mM ascorbate and 1% (v/v) Triton X-100. After centrifugation at 3500×g for 10 min in a microcentrifuge at 4°C, the supernatants were used to determine enzyme activity and protein content [66]. The activities of SOD, APX, and CAT were analyzed as proposed by Beauchamp and Fridovich [67], Nakano and Asada [68], and Aebi [69], respectively. Six plants per line (total 15 lines and WT) were evaluated. Trials were repeated three times. Data were analyzed by one-way ANOVA in SAS [70]. Means of enzymes were compared using Fisher’s least significant difference (LSD) test [70].

Chloroplasts were isolated using the Chloroplast Isolation Kit (P 4937; Sigma-Aldrich, St. Louis, MO, USA) with minor modification of the recommended protocol. The fully-expanded young leaves were kept in the dark overnight before isolating the chloroplasts, and the leaf homogenate was centrifuged for 3 min at 250×g. The activities of SOD, APX, and CAT in chloroplasts were assayed as described above.

### Analysis of Transgenic Plant Tolerance to MV Stress

To investigate the tolerances of 15 transgenic lines to MV-induced oxidative stress, we carried out trials of leaf discs and of whole plants sprayed with MV solution. Twelve leaf discs (1 cm diam.) were collected from each 30-d-old transgenic and WT greenhouse plant and treated with 5 µM MV solution. Four plants per line were employed. The extent of cellular damage was assessed by leaf injury after 36 h under continuous light (200 µmol m^−2 ^s^−1^) at 28°C. Cellular damage in MV-treated leaf discs was also assessed by electrolyte leakage over 48 h [19]. At the whole plant level, the 30-d-old plants (15–20 cm high) of each line (6 plants per line) were sprayed with 60 ml of 300 µM MV solution. We evaluated the visible damage on the leaves 5 d after treatment on a scale from 0–100 (0, no damage; 100, fully-damage leaves). In addition, total leaf chlorophyll content was measured 5 d after MV treatment [71]. Each trial was repeated three times. Data were analyzed by one-way ANOVA using SAS [70]. Means of membrane ion leakage, visible injury, and total chlorophyll were compared using LSD [70].

### Analysis of Transgenic Plant Tolerance to Salt Stress

The salt tolerance test was carried out as proposed by He et al [52] with minor modifications. Seeds of WT and 15 transgenic lines were planted individually into soil in 30 cm-diam. pots in the greenhouse. Two weeks later, incremental salt treatment was initiated, starting with 50 mM NaCl for 7 d followed by 7 d each of 100 and 150 mM. Finally, 200 mM NaCl was applied for 21 d or until the end of the experiment. The temperature in the greenhouse was maintained at 28/25°C (day/night), and the relative humidity was maintained at 50±10%. Six plants per line were employed, and each trial was repeated three times. The control group for each line was watered with tap water.

### Biomass Measurements and Fiber Yield under Salt Treatment

Shoots (above-ground portion) and roots (under-ground portion) were each harvested for fresh biomass determination after 4 wks of 200 mM NaCl treatment in the greenhouse, as described above. Roots were harvested by gently flushing soil away with water and drying with paper. Plant height, bolls per plant, and cotton fiber yield (with seeds) was determined when all bolls had opened and matured in salt-treated and control plants. The photosynthetic efficiency of leaves after 2 d of NaCl treatment, which seemed to cause wilt by this time, was measured by chlorophyll fluorescence determination of photochemical yield (*Fv/Fm*) [46]. Six plants per line were employed, with three replicates per evaluation. The data were analyzed by one-way ANOVA using SAS [70]. Means of fresh root weight, fresh shoot weight, plant height, bolls per plant, and cotton fiber yield (with seeds) were compared using LSD [70].

## Supporting Information

Figure S1 **RT-PCR expression profiles of the** ***GhSOD1, GhAPX1,*** **and** ***GhCAT1*** **genes in cotton tissues.** *GhUBI* gene was used as an internal control.
(TIF)

Figure S2 **PCR and semi-quantitative RT-PCR analysis of transgenic cotton plants.** (A) PCR analysis of *GhSOD1, GhAPX1,* or *GhCAT1* in three types of transgenic cotton plants. M, DNA Marker DL2000; WT, WT plant; P, plasmid with target gene; 1–8, representative transgenic plants for each target gene. (B) Analysis of overexpression of *GhSOD1, GhAPX1,* or *GhCAT1* in six representative transgenic plants using semi-quantitative RT-PCR. *GhUBI* was used as a control for normalization. Lanes as in (A).
(TIF)

Figure S3 **Tolerance of two types of transgenic cotton plant to salt stress.** Activities of SOD (A) and CAT (B) enzymes in transgenic plants overexpressing both genes in the cytoplasm (SCT-p) or chloroplast (SCT-c). (C) Phenotypes of WT and transgenic cotton plants 21 d after treatment with 200 mM NaCl. Values are given as means ± standard deviation.
(TIF)

Table S1 The primer sets used for isolation of target genes, PCR, and RT-PCR.
(DOC)

Table S2 Agronomic traits of transgenic and WT plants after treatment with tap water in the greenhouse as a control group. Three lines every kind of transgenic cotton, 6 plants per line, 18 plants in WT; experiment was repeated at three times. Values are given as means ± standard deviation. (n = 54). Means within a column followed by different letters are significantly different at *P*<0.05.
(DOC)
