# Supplementary figures and images for: Synergistic Effects of GhSOD1 and GhCAT1 Overexpression in Cotton Chloroplasts on Enhancing Tolerance to Methyl Viologen and Salt Stresses
Source: PLoS One. 2013 Jan 15;8(1):e54002. doi: 10.1371/journal.pone.0054002 (PMC3545958; doi:10.1371/journal.pone.0054002)

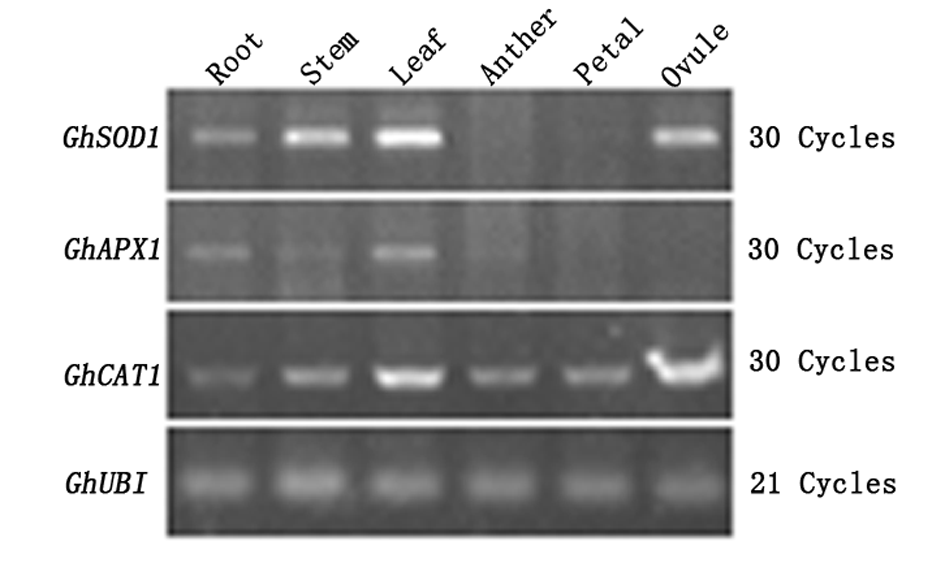

Supplement: Figure S1 — RT-PCR expression profiles of the GhSOD1, GhAPX1, and GhCAT1 genes in cotton tissues. GhUBI gene was used as an internal control. (TIF) [file pone.0054002.s001.tif]

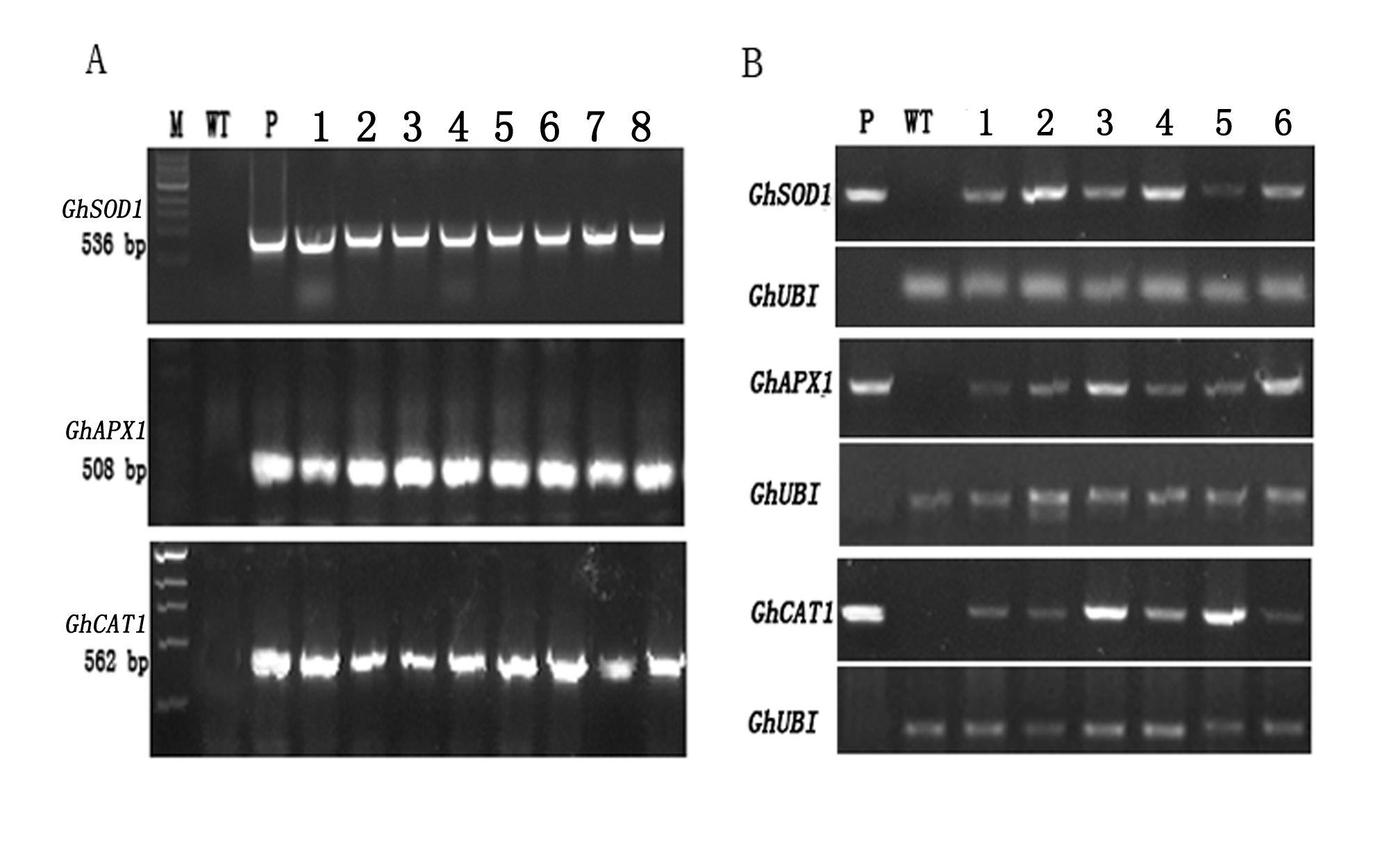

Supplement: Figure S2 — PCR and semi-quantitative RT-PCR analysis of transgenic cotton plants. (A) PCR analysis of GhSOD1, GhAPX1, or GhCAT1 in three types of transgenic cotton plants. M, DNA Marker DL2000; WT, WT plant; P, plasmid with target gene; 1–8, representative transgenic plants for each target gene. (B) Analysis of overexpression of GhSOD1, GhAPX1, or GhCAT1 in six representative transgenic plants using semi-quantitative RT-PCR. GhUBI was used as a control for normalization. Lanes as in (A). (TIF) [file pone.0054002.s002.tif]

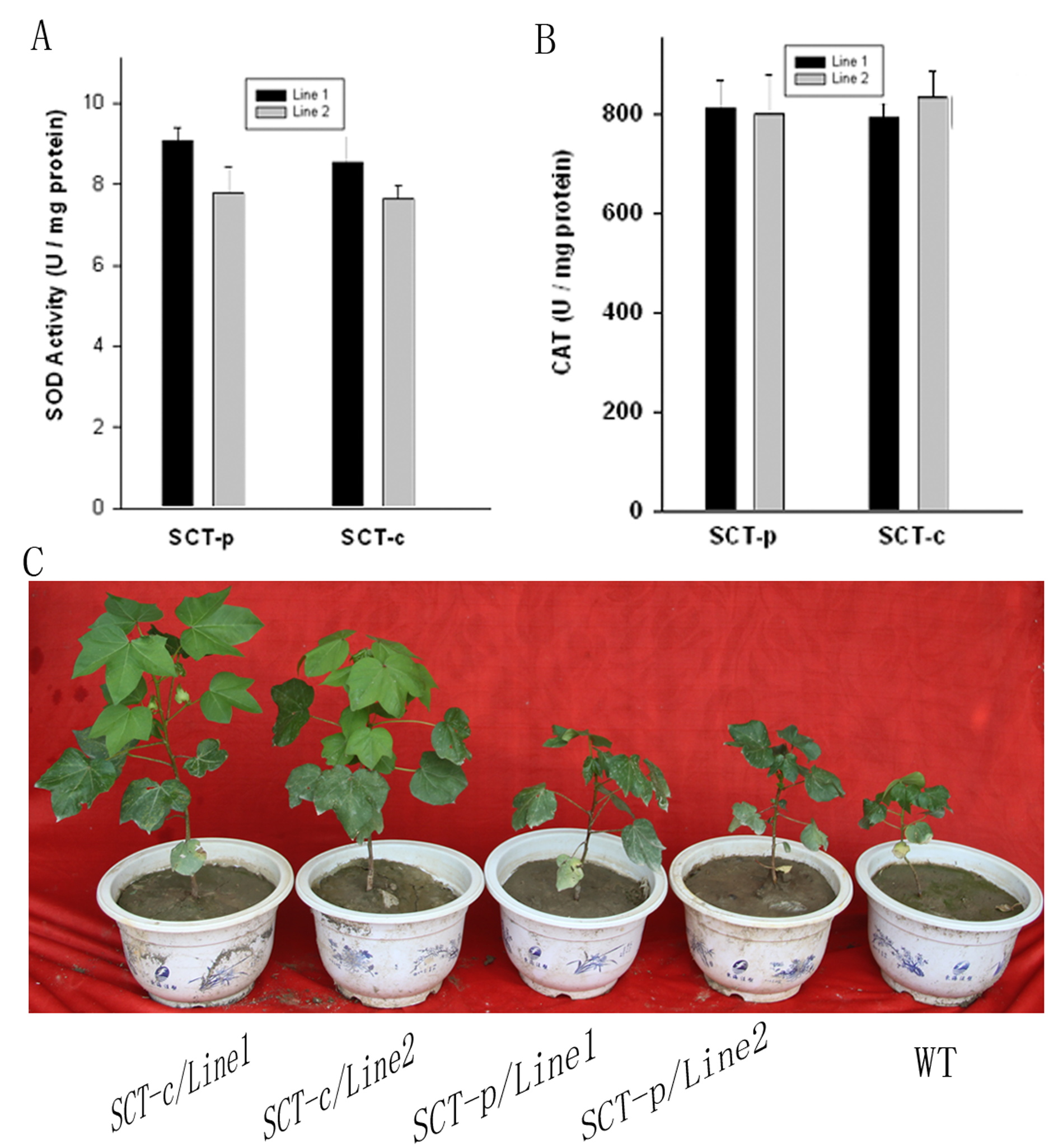

Supplement: Figure S3 — Tolerance of two types of transgenic cotton plant to salt stress. Activities of SOD (A) and CAT (B) enzymes in transgenic plants overexpressing both genes in the cytoplasm (SCT-p) or chloroplast (SCT-c). (C) Phenotypes of WT and transgenic cotton plants 21 d after treatment with 200 mM NaCl. Values are given as means ± standard deviation. (TIF) [file pone.0054002.s003.tif]
