# Supplementary material for: Synergistic Effects of GhSOD1 and GhCAT1 Overexpression in Cotton Chloroplasts on Enhancing Tolerance to Methyl Viologen and Salt Stresses
Source: PLoS One. 2013 Jan 15;8(1):e54002. doi: 10.1371/journal.pone.0054002 (PMC3545958; doi:10.1371/journal.pone.0054002)
Supplement: Table S1 — The primer sets used for isolation of target genes, PCR, and RT-PCR. (DOC) [file pone.0054002.s004.doc]

Table S1. Primer sets used to isolate target genes and for PCR and RT-PCR

| Genes | Forward | Reverse |
| --- | --- | --- |
| For gene isolation | | |
| *GhSOD1* | atggctaaggccgttgctg | tcacccttgcagaccaataa |
| *GhAPX1* | atgggaaccaagtgttac | ttatgcatcagcaaatcc |
| *GhCAT1* | atggatccctacaagcacc | ctaaatgctgggtctcacattg |
| For PCR and RT-PCR analysis | | |
| *GhSOD1* | gacgagaaggtacatgacg | cagcatggacaacgaccgc |
| *GhAPX1* | gacgagaaggtacatgacg | cttcccagagtgtccgcc |
| *GhCAT1* | gacgagaaggtacatgacg | agagcatggaccatatcag |
| *GhUBI* | ctgaatcttcgctttcacgttatc | gggatgcaaatcttcgttaagac |
| For qRT-PCR analysis | | |
| *GhSOD1* | catctctcacgcactctgtc | ccttagccatttctgtctgtg |
| *GhAPX1* | tcgttgccgttgagattac | tggtagcatcaggaagacg |
| *GhCAT1* | tgataagttgctccagactcg | cttcgtggtgattgttgtga |
| *GhUBI* | gagacgtagttagaaaggaag | agtacgttcccattccggaac |
