# Supplementary material for: Synergistic Effects of GhSOD1 and GhCAT1 Overexpression in Cotton Chloroplasts on Enhancing Tolerance to Methyl Viologen and Salt Stresses
Source: PLoS One. 2013 Jan 15;8(1):e54002. doi: 10.1371/journal.pone.0054002 (PMC3545958; doi:10.1371/journal.pone.0054002)
Supplement: Table S2 — Agronomic traits of transgenic and WT plants after treatment with tap water in the greenhouse as a control group. Three lines every kind of transgenic cotton, 6 plants per line, 18 plants in WT; experiment was repeated at three times. Values are given as means ± standard deviation. (n = 54). Means within a column followed by different letters are significantly different at P<0.05. (DOC) [file pone.0054002.s005.doc]

**Table S2. Agronomic traits of transgenic and WT cotton plants treated with tap water as a control.** Three lines of each of five kinds of transgenic cotton (six plants per line, 18 WT plants) were treated; the experiment was repeated three times. Values are given as means ± standard deviation (n=54). Means within a column followed by different letters are significantly different at *P* < 0.05.

| Lines | Plant height (cm) | Bolls per plant | Boll weight (g) | Seed fiber yields per plant (g) |
| --- | --- | --- | --- | --- |
| WT | 81.6±6.5 a | 8.6±0.6 a | 5.0±0.4 a | 44.1±3.0 a |
| ST | 81.9±6.3 a | 8.4±0.7 a | 4.8±0.3 a | 40.9±3.1 a |
| AT | 84.5±7.2 a | 8.8±0.7 a | 4.9±0.5 a | 43.8±2.8 a |
| CT | 89.8±6.2 a | 8.7±0.8 a | 4.9±0.4 a | 43.1±2.9 a |
| SAT | 85.5±8.1 a | 8.9±0.7 b | 4.8±0.3 a | 44.2±3.5 a |
| SCT | 88.5±7.6 a | 8.8±0.7 a | 4.9±0.4 a | 43.9±3.1 a |
